# Chlorido{*N*-[2-(diphenyl­phosphan­yl)benzyl­idene]-2,6-diisopropyl­aniline-κ*P*}gold(I) chloro­form 0.25-solvate

**DOI:** 10.1107/S1600536812049975

**Published:** 2012-12-12

**Authors:** Haleden Chiririwa, Wade L. Davis

**Affiliations:** a Department of Chemistry, University of Cape Town, Private Bag, Rondebosch, 7707, South Africa; b Research Centre for Synthesis and Catalysis, Department of Chemistry, University of Johannesburg (APK Campus), PO Box 524, Auckland Park, Johannesburg, 2006, South Africa

## Abstract

The asymmetric unit of the title compound, [AuCl(C_31_H_32_NP)]·0.25CHCl_3_, contains two independent complex mol­ecules and half a chloro­form solvent mol­ecule, which is disordered across an inversion center. The Au^I^ ions are each coordinated in a slightly distorted linear environment, with P—Au—Cl angles of 177.20 (4) and 178.54 (4)°.

## Related literature

For general background to gold complexes, see: Shaw (1999 ▶); Barnard *et al.* (2004 ▶); Nomiya *et al.* (2003 ▶). For applications of gold-containing drugs, see: Chiririwa *et al.* (2013 ▶); Fricker (1996 ▶); Cowan (1993 ▶); Parish (1992 ▶); Finkelstein *et al.* (1976 ▶). For the synthesis of the starting materials, see: Mogorosi *et al.* (2011 ▶); Uson & Laguna (1986 ▶); Reddy *et al.* (2002 ▶). For similar compounds, see: Chiririwa & Muller (2012 ▶); Williams *et al.* (2007 ▶).
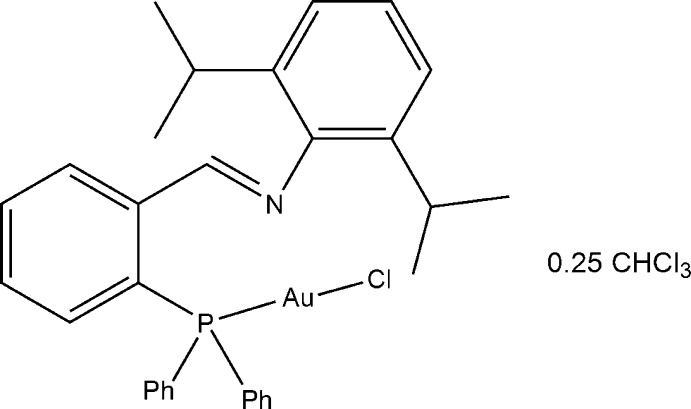


## Experimental

### 

#### Crystal data

[AuCl(C_31_H_32_NP)]·0.25CHCl_3_
*M* *_r_* = 711.81
Triclinic, 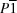

*a* = 13.0315 (3) Å
*b* = 13.3638 (2) Å
*c* = 19.3489 (5) Å
α = 96.358 (2)°
β = 99.229 (1)°
γ = 116.191 (1)°
*V* = 2921.15 (11) Å^3^
*Z* = 4
Mo *K*α radiation
μ = 5.27 mm^−1^
*T* = 173 K
0.21 × 0.18 × 0.12 mm

#### Data collection

Bruker APEXII 4K CCD diffractometer
Absorption correction: multi-scan (*SADABS*; Bruker, 2007 ▶) *T*_min_ = 0.404, *T*_max_ = 0.570
119407 measured reflections
11945 independent reflections
9894 reflections with *I* > 2σ(*I*)
*R*_int_ = 0.053

#### Refinement

*R*[*F* ^2^ > 2σ(*F* ^2^)] = 0.030
*wR*(*F* ^2^) = 0.065
*S* = 1.09
11945 reflections
667 parameters
H-atom parameters constrained
Δρ_max_ = 1.91 e Å^−3^
Δρ_min_ = −1.54 e Å^−3^

### 

Data collection: *APEX2* (Bruker, 2007 ▶); cell refinement: *SAINT* (Bruker, 2007 ▶); data reduction: *SAINT* and *XPREP* (Bruker, 2007 ▶); program(s) used to solve structure: *SHELXS97* (Sheldrick, 2008 ▶); program(s) used to refine structure: *SHELXL97* (Sheldrick, 2008 ▶); molecular graphics: *DIAMOND* (Brandenburg & Putz, 2005 ▶); software used to prepare material for publication: *WinGX* (Farrugia, 2012) ▶.

## Supplementary Material

Crystal structure: contains datablock(s) I, global. DOI: 10.1107/S1600536812049975/lh5564sup1.cif

Structure factors: contains datablock(s) I. DOI: 10.1107/S1600536812049975/lh5564Isup2.hkl

Additional supplementary materials:  crystallographic information; 3D view; checkCIF report

## Figures and Tables

**Fig. 1.**
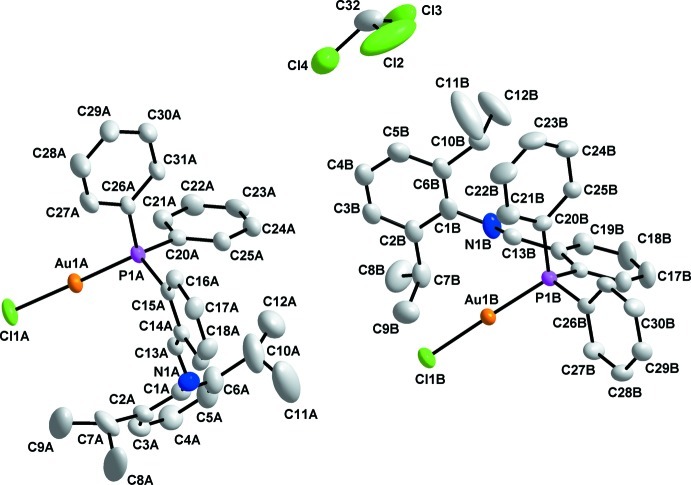
Molecular structure of the title complex, showing 50% probability displacement ellipsoids. The chloroform solvent molecule is half occupancy. There are two crystallographic independent molecules labelled as A and B in the asymmetric unit. All hydrogen atoms are omitted for clarity.

## supplementary crystallographic information

### Comment

Studies of gold(I) complexes have been related to anti-arthritic (Shaw, 1999), anti-tumor (Barnard *et al.*, 2004) and antimicrobial physiological activities (Nomiya *et al.*, 2003). The treatment of diseases with gold complexes (called chrysotherapy) can be traced back to early times (Cowan, 1993). The mechanistic approach regarding the action of gold drugs is still a matter of interest and research, however they remain the most effective second line treatment for rheumatoid arthritis (Parish, 1992; Fricker, 1996). This led to the development of a gold complex called auranofin, which was found to be an experimental chrysotherapeutic agent and was clinically effective in the treatment of rheumatoid arthritis (Finkelstein *et al.*, 1976). Our group has recently been involved in the development of novel Au(I) complexes with iminophosphine ligands and these have shown promising activity when evaluated against two oesophageal cancer cell lines (Chiririwa *et al.*, 2013).

The structure of the title compound (Fig. 1) was determined to establish the coordination properties of the 2-(diphenylphosphanyl)benzylidene)- 2,6-diisopropylbenzenamine ligand with gold. The asymmetric unit consists of two crystallographically independent complex molecules. The Au—P bond distances of 2.2354 (10) and 2.2267 (10) Å agree well with reported values (Williams *et al.*., 2007; Chiririwa & Muller, 2012). The chloroform solvent molecule is disordered across an inversion centre.

### Experimental

The iminophosphine ligand was synthesized in high yields from the Schiff-base condensation reaction of 2-(diphenylphosphanyl)benzaldehyde with 2,6-diisopropylaniline in CH_2_Cl_2_ at room temperature (Mogorosi *et al.*, 2011; Reddy *et al.*, 2002). The Au precursor was synthesized by slow drop wise addition of tetrahydrothiophene (THT) to a solution of HAuCl_4_.4H_2_O in EtOH (Uson & Laguna, 1986). To a dry CH_2_Cl_2_ (10 ml) solution of the precursor [Au(tht)Cl] (tht = tetrahydrothiophene) was added an equimolar amount of 2-(diphenylphosphanyl)benzylidene)-2,6 diisopropylbenzenamine in CH_2_Cl_2_ (10 ml), and stirred at room temperature for 2 hrs. The solvent was reduced under reduced pressure and on addition of hexane, the product was filtered off and washed with Et_2_O (2 × 5 ml) and dried under vacuum for 4 hrs affording a yellow precipitate. Crystals suitable for X-ray structure determination were obtained by recrystallization from a CHCl_3_/hexane mixture at room temperature. ^31^P NMR: 26.64 (*s*). IR (KBr): 1628 cm^-1^ (C=N, imine).

### Refinement

All H atoms were included in calculated positions with C—H = 0.95–1.00 Å and included in the refinement with *U*_iso_(H) = 1.2*U*_eq_(C) or 1.5 *U*_eq_(C_methyl_). The chloroform solvate molecule is disordered over an inversion centre, H atom connectivity was correctly assigned by using a PART -1 instruction in *SHELXL97* (Sheldrick, 2008). Occupancies of each disordered component was constrained to 50% conforming to the imposed crystallographic symmetry. No additional geometrical or thermal ellipsoid restraints were employed in the final refinement cycles.

### Crystal data

**Table:**

[AuCl(C_31_H_32_NP)]·0.25CHCl_3_	*Z* = 4
*M**_r_* = 711.81	*F*(000) = 1402
Triclinic, *P*1	*D*_x_ = 1.619 Mg m^−^^3^
Hall symbol: -P 1	Mo *K*α radiation, λ = 0.71073 Å
*a* = 13.0315 (3) Å	Cell parameters from 119370 reflections
*b* = 13.3638 (2) Å	θ = 2.3–26.4°
*c* = 19.3489 (5) Å	µ = 5.27 mm^−^^1^
α = 96.358 (2)°	*T* = 173 K
β = 99.229 (1)°	Block, yellow
γ = 116.191 (1)°	0.21 × 0.18 × 0.12 mm
*V* = 2921.15 (11) Å^3^	

### Data collection

**Table:**

Bruker APEXII 4K CCD diffractometer	11945 independent reflections
Radiation source: fine-focus sealed tube	9894 reflections with *I* > 2σ(*I*)
Graphite monochromator	*R*_int_ = 0.053
Detector resolution: 0 pixels mm^-1^	θ_max_ = 26.4°, θ_min_ = 1.8°
0.5° ω scans, 20s	*h* = −16→16
Absorption correction: multi-scan (*SADABS*; Bruker, 2007)	*k* = −16→16
*T*_min_ = 0.404, *T*_max_ = 0.570	*l* = −24→24
119407 measured reflections	

### Refinement

**Table:**

Refinement on *F*^2^	Primary atom site location: structure-invariant direct methods
Least-squares matrix: full	Secondary atom site location: difference Fourier map
*R*[*F*^2^ > 2σ(*F*^2^)] = 0.030	Hydrogen site location: inferred from neighbouring sites
*wR*(*F*^2^) = 0.065	H-atom parameters constrained
*S* = 1.09	*w* = 1/[σ^2^(*F*_o_^2^) + (0.0042*P*)^2^ + 7.7368*P*] where *P* = (*F*_o_^2^ + 2*F*_c_^2^)/3
11945 reflections	(Δ/σ)_max_ = 0.001
667 parameters	Δρ_max_ = 1.91 e Å^−^^3^
0 restraints	Δρ_min_ = −1.54 e Å^−^^3^

### Special details

**Table:**

Geometry. All e.s.d.'s (except the e.s.d. in the dihedral angle between two l.s. planes) are estimated using the full covariance matrix. The cell e.s.d.'s are taken into account individually in the estimation of e.s.d.'s in distances, angles and torsion angles; correlations between e.s.d.'s in cell parameters are only used when they are defined by crystal symmetry. An approximate (isotropic) treatment of cell e.s.d.'s is used for estimating e.s.d.'s involving l.s. planes.
Refinement. Refinement of *F*^2^ against ALL reflections. The weighted *R*-factor *wR* and goodness of fit *S* are based on *F*^2^, conventional *R*-factors *R* are based on *F*, with *F* set to zero for negative *F*^2^. The threshold expression of *F*^2^ > σ(*F*^2^) is used only for calculating *R*-factors(gt) *etc*. and is not relevant to the choice of reflections for refinement. *R*-factors based on *F*^2^ are statistically about twice as large as those based on *F*, and *R*- factors based on ALL data will be even larger.

### Fractional atomic coordinates and isotropic or equivalent isotropic displacement parameters (Å^2^)

**Table:**

	*x*	*y*	*z*	*U*_iso_*/*U*_eq_	Occ. (<1)
Au1A	0.261779 (14)	−0.032391 (14)	−0.065469 (8)	0.03058 (5)	
Cl1A	0.21007 (11)	−0.10467 (11)	−0.18529 (6)	0.0472 (3)	
P1A	0.31961 (9)	0.03622 (9)	0.05254 (5)	0.0273 (2)	
N1A	0.3385 (3)	0.3431 (3)	−0.0152 (2)	0.0425 (10)	
C1A	0.2277 (4)	0.3255 (4)	−0.0571 (2)	0.0371 (10)	
C2A	0.2036 (4)	0.2940 (4)	−0.1316 (2)	0.0364 (10)	
C3A	0.1033 (4)	0.2912 (4)	−0.1706 (3)	0.0453 (12)	
H3A	0.0857	0.2723	−0.2214	0.054*	
C4A	0.0285 (5)	0.3153 (5)	−0.1376 (3)	0.0545 (14)	
H4A	−0.0395	0.3134	−0.1656	0.065*	
C5A	0.0515 (5)	0.3418 (5)	−0.0643 (3)	0.0545 (14)	
H5A	−0.0017	0.3567	−0.0420	0.065*	
C6A	0.1513 (4)	0.3473 (5)	−0.0222 (3)	0.0516 (13)	
C7A	0.2839 (5)	0.2630 (6)	−0.1691 (3)	0.0581 (15)	
H7A	0.3212	0.2312	−0.1348	0.070*	
C8A	0.3811 (7)	0.3648 (6)	−0.1823 (5)	0.109 (3)	
H8AA	0.4306	0.3420	−0.2060	0.163*	
H8AB	0.4284	0.4175	−0.1366	0.163*	
H8AC	0.3486	0.4027	−0.2129	0.163*	
C9A	0.2199 (7)	0.1711 (6)	−0.2367 (4)	0.101 (3)	
H9AA	0.1552	0.1052	−0.2266	0.152*	
H9AB	0.2749	0.1478	−0.2532	0.152*	
H9AC	0.1883	0.2009	−0.2739	0.152*	
C10A	0.1770 (6)	0.3745 (7)	0.0610 (3)	0.088 (2)	
H10A	0.2204	0.3334	0.0792	0.106*	
C11A	0.2554 (10)	0.4963 (9)	0.0867 (5)	0.182 (6)	
H11D	0.3256	0.5183	0.0674	0.273*	
H11E	0.2786	0.5131	0.1391	0.273*	
H11F	0.2145	0.5391	0.0710	0.273*	
C12A	0.0687 (8)	0.3357 (7)	0.0899 (4)	0.100 (3)	
H12D	0.0156	0.2550	0.0688	0.150*	
H12E	0.0288	0.3809	0.0778	0.150*	
H12F	0.0908	0.3458	0.1420	0.150*	
C13A	0.3428 (4)	0.2607 (4)	0.0059 (2)	0.0348 (10)	
H13A	0.2735	0.1893	−0.0062	0.042*	
C14A	0.4531 (3)	0.2695 (4)	0.0498 (2)	0.0280 (9)	
C15A	0.4521 (3)	0.1752 (3)	0.0765 (2)	0.0267 (9)	
C16A	0.5551 (3)	0.1859 (4)	0.1187 (2)	0.0307 (9)	
H16A	0.5548	0.1230	0.1374	0.037*	
C17A	0.6582 (4)	0.2884 (4)	0.1334 (2)	0.0371 (11)	
H17A	0.7282	0.2953	0.1621	0.044*	
C18A	0.6599 (4)	0.3801 (4)	0.1066 (3)	0.0417 (11)	
H18A	0.7308	0.4498	0.1164	0.050*	
C19A	0.5574 (4)	0.3704 (4)	0.0651 (2)	0.0359 (10)	
H19A	0.5588	0.4340	0.0469	0.043*	
C20A	0.2136 (3)	0.0562 (3)	0.0959 (2)	0.0288 (9)	
C21A	0.0961 (4)	−0.0217 (4)	0.0732 (2)	0.0375 (11)	
H21A	0.0700	−0.0828	0.0337	0.045*	
C22A	0.0160 (4)	−0.0103 (4)	0.1083 (3)	0.0446 (12)	
H22A	−0.0651	−0.0640	0.0928	0.054*	
C23A	0.0530 (4)	0.0783 (4)	0.1654 (3)	0.0428 (12)	
H23A	−0.0025	0.0857	0.1891	0.051*	
C24A	0.1711 (4)	0.1566 (4)	0.1885 (2)	0.0371 (10)	
H24A	0.1970	0.2176	0.2282	0.045*	
C25A	0.2513 (4)	0.1454 (4)	0.1533 (2)	0.0322 (10)	
H25A	0.3323	0.1992	0.1687	0.039*	
C26A	0.3579 (3)	−0.0571 (3)	0.0989 (2)	0.0290 (9)	
C27A	0.4052 (5)	−0.1184 (4)	0.0658 (3)	0.0465 (12)	
H27A	0.4157	−0.1116	0.0187	0.056*	
C28A	0.4372 (6)	−0.1890 (5)	0.0999 (3)	0.0604 (16)	
H28A	0.4719	−0.2287	0.0771	0.073*	
C29A	0.4188 (5)	−0.2016 (4)	0.1671 (3)	0.0534 (14)	
H29A	0.4393	−0.2515	0.1903	0.064*	
C30A	0.3709 (4)	−0.1429 (4)	0.2010 (3)	0.0461 (12)	
H30A	0.3582	−0.1523	0.2474	0.055*	
C31A	0.3413 (4)	−0.0699 (4)	0.1674 (2)	0.0402 (11)	
H31A	0.3095	−0.0282	0.1912	0.048*	
Au1B	0.513749 (14)	0.777609 (13)	0.449875 (8)	0.02904 (5)	
Cl1B	0.50551 (13)	0.74084 (11)	0.33113 (6)	0.0508 (3)	
P1B	0.52319 (9)	0.81829 (9)	0.56620 (5)	0.0251 (2)	
N1B	0.1566 (3)	0.5623 (3)	0.4766 (2)	0.0439 (10)	
C1B	0.1405 (4)	0.4560 (4)	0.4387 (2)	0.0387 (11)	
C2B	0.0953 (4)	0.4264 (4)	0.3643 (2)	0.0385 (11)	
C3B	0.0748 (4)	0.3211 (4)	0.3282 (3)	0.0420 (12)	
H3B	0.0450	0.3002	0.2778	0.050*	
C4B	0.0964 (5)	0.2467 (4)	0.3637 (3)	0.0533 (14)	
H4B	0.0822	0.1753	0.3380	0.064*	
C5B	0.1392 (5)	0.2762 (4)	0.4374 (3)	0.0544 (14)	
H5B	0.1538	0.2240	0.4615	0.065*	
C6B	0.1614 (4)	0.3803 (4)	0.4770 (2)	0.0452 (13)	
C7B	0.0733 (4)	0.5084 (4)	0.3238 (3)	0.0469 (12)	
H7B	0.0749	0.5696	0.3595	0.056*	
C8B	−0.0477 (5)	0.4502 (5)	0.2709 (4)	0.079 (2)	
H8BA	−0.0584	0.5062	0.2460	0.118*	
H8BB	−0.1096	0.4188	0.2970	0.118*	
H8BC	−0.0523	0.3886	0.2359	0.118*	
C9B	0.1719 (5)	0.5645 (5)	0.2862 (3)	0.0637 (16)	
H9BA	0.1573	0.6176	0.2602	0.096*	
H9BB	0.1747	0.5059	0.2525	0.096*	
H9BC	0.2471	0.6062	0.3217	0.096*	
C10B	0.1994 (5)	0.4048 (4)	0.5582 (3)	0.0537 (14)	
H10B	0.2425	0.4893	0.5757	0.064*	
C11B	0.2786 (6)	0.3564 (9)	0.5861 (4)	0.124 (4)	
H11A	0.3493	0.3872	0.5671	0.185*	
H11B	0.2368	0.2733	0.5710	0.185*	
H11C	0.3012	0.3773	0.6385	0.185*	
C12B	0.0944 (6)	0.3580 (8)	0.5893 (3)	0.093 (3)	
H12A	0.1198	0.3751	0.6416	0.140*	
H12B	0.0499	0.2753	0.5719	0.140*	
H12C	0.0442	0.3928	0.5748	0.140*	
C13B	0.2586 (3)	0.6349 (4)	0.5107 (2)	0.0300 (9)	
H13B	0.3228	0.6203	0.5070	0.036*	
C14B	0.2817 (3)	0.7428 (4)	0.5567 (2)	0.0285 (9)	
C15B	0.3963 (3)	0.8319 (3)	0.5849 (2)	0.0255 (9)	
C16B	0.4137 (4)	0.9304 (4)	0.6290 (2)	0.0358 (10)	
H16B	0.4913	0.9910	0.6475	0.043*	
C17B	0.3193 (4)	0.9413 (5)	0.6464 (3)	0.0498 (13)	
H17B	0.3319	1.0087	0.6765	0.060*	
C18B	0.2073 (4)	0.8532 (5)	0.6193 (3)	0.0522 (14)	
H18B	0.1424	0.8598	0.6314	0.063*	
C19B	0.1877 (4)	0.7551 (4)	0.5748 (2)	0.0408 (11)	
H19B	0.1096	0.6956	0.5562	0.049*	
C20B	0.5325 (4)	0.7150 (3)	0.6169 (2)	0.0303 (9)	
C21B	0.5863 (4)	0.6517 (4)	0.5954 (3)	0.0472 (12)	
H21B	0.6147	0.6601	0.5530	0.057*	
C22B	0.5986 (5)	0.5763 (5)	0.6355 (3)	0.0583 (15)	
H22B	0.6360	0.5335	0.6206	0.070*	
C23B	0.5574 (5)	0.5629 (4)	0.6964 (3)	0.0538 (14)	
H23B	0.5659	0.5107	0.7234	0.065*	
C24B	0.5036 (4)	0.6251 (4)	0.7184 (3)	0.0441 (12)	
H24B	0.4752	0.6157	0.7607	0.053*	
C25B	0.4908 (4)	0.7011 (4)	0.6794 (2)	0.0335 (10)	
H25B	0.4537	0.7438	0.6949	0.040*	
C26B	0.6515 (3)	0.9531 (3)	0.6079 (2)	0.0259 (9)	
C27B	0.6739 (4)	1.0461 (4)	0.5752 (2)	0.0360 (10)	
H27B	0.6222	1.0376	0.5318	0.043*	
C28B	0.7708 (4)	1.1509 (4)	0.6056 (2)	0.0392 (11)	
H28B	0.7845	1.2144	0.5836	0.047*	
C29B	0.8472 (4)	1.1626 (4)	0.6678 (2)	0.0376 (11)	
H29B	0.9138	1.2343	0.6886	0.045*	
C30B	0.8276 (4)	1.0710 (4)	0.7001 (2)	0.0380 (11)	
H30B	0.8812	1.0795	0.7426	0.046*	
C31B	0.7295 (4)	0.9662 (4)	0.6705 (2)	0.0324 (10)	
H31B	0.7157	0.9034	0.6932	0.039*	
Cl4	0.0094 (7)	−0.0310 (5)	0.4177 (3)	0.125 (2)	0.50
Cl3	−0.0785 (7)	0.0091 (5)	0.5291 (5)	0.152 (3)	0.50
Cl2	0.1757 (5)	0.0690 (5)	0.5517 (5)	0.196 (4)	0.50
C32	0.0374 (12)	−0.0182 (11)	0.5187 (8)	0.073 (4)	0.50
H32	0.0202	−0.0946	0.5297	0.087*	0.50

### Atomic displacement parameters (Å^2^)

**Table:**

	*U*^11^	*U*^22^	*U*^33^	*U*^12^	*U*^13^	*U*^23^
Au1A	0.03151 (9)	0.03684 (10)	0.02132 (9)	0.01715 (8)	0.00154 (7)	−0.00024 (7)
Cl1A	0.0554 (7)	0.0701 (8)	0.0226 (6)	0.0421 (7)	−0.0012 (5)	−0.0063 (5)
P1A	0.0270 (5)	0.0312 (6)	0.0202 (5)	0.0126 (5)	0.0029 (4)	0.0010 (4)
N1A	0.040 (2)	0.040 (2)	0.041 (2)	0.0153 (19)	0.0033 (18)	0.0086 (19)
C1A	0.033 (2)	0.040 (3)	0.037 (3)	0.019 (2)	0.001 (2)	0.008 (2)
C2A	0.036 (2)	0.043 (3)	0.031 (2)	0.018 (2)	0.0076 (19)	0.013 (2)
C3A	0.047 (3)	0.051 (3)	0.033 (3)	0.020 (2)	0.000 (2)	0.014 (2)
C4A	0.048 (3)	0.066 (4)	0.052 (3)	0.035 (3)	−0.006 (3)	0.010 (3)
C5A	0.046 (3)	0.067 (4)	0.058 (4)	0.038 (3)	0.008 (3)	0.002 (3)
C6A	0.046 (3)	0.066 (4)	0.041 (3)	0.032 (3)	−0.001 (2)	−0.005 (3)
C7A	0.051 (3)	0.102 (5)	0.029 (3)	0.040 (3)	0.012 (2)	0.018 (3)
C8A	0.112 (6)	0.069 (5)	0.141 (7)	0.025 (4)	0.089 (6)	−0.007 (5)
C9A	0.109 (6)	0.065 (4)	0.125 (7)	0.030 (4)	0.064 (5)	−0.008 (4)
C10A	0.078 (5)	0.143 (7)	0.056 (4)	0.079 (5)	−0.006 (3)	−0.026 (4)
C11A	0.185 (11)	0.134 (9)	0.090 (7)	−0.037 (8)	0.057 (7)	−0.044 (6)
C12A	0.145 (8)	0.095 (6)	0.063 (5)	0.053 (5)	0.036 (5)	0.022 (4)
C13A	0.050 (3)	0.038 (3)	0.024 (2)	0.025 (2)	0.012 (2)	0.0074 (19)
C14A	0.029 (2)	0.037 (2)	0.021 (2)	0.0189 (19)	0.0047 (17)	0.0045 (18)
C15A	0.028 (2)	0.034 (2)	0.019 (2)	0.0159 (18)	0.0058 (16)	0.0006 (17)
C16A	0.029 (2)	0.035 (2)	0.029 (2)	0.0179 (19)	0.0021 (18)	0.0051 (19)
C17A	0.023 (2)	0.040 (3)	0.040 (3)	0.013 (2)	−0.0037 (19)	−0.002 (2)
C18A	0.031 (2)	0.034 (3)	0.048 (3)	0.008 (2)	0.003 (2)	0.001 (2)
C19A	0.041 (3)	0.031 (2)	0.036 (3)	0.018 (2)	0.007 (2)	0.0038 (19)
C20A	0.028 (2)	0.032 (2)	0.026 (2)	0.0142 (19)	0.0024 (17)	0.0091 (18)
C21A	0.032 (2)	0.040 (3)	0.034 (3)	0.013 (2)	0.0037 (19)	0.005 (2)
C22A	0.027 (2)	0.054 (3)	0.046 (3)	0.012 (2)	0.008 (2)	0.015 (2)
C23A	0.040 (3)	0.060 (3)	0.047 (3)	0.032 (3)	0.023 (2)	0.025 (3)
C24A	0.041 (3)	0.043 (3)	0.034 (3)	0.024 (2)	0.012 (2)	0.008 (2)
C25A	0.028 (2)	0.038 (2)	0.029 (2)	0.0146 (19)	0.0076 (18)	0.0051 (19)
C26A	0.029 (2)	0.033 (2)	0.022 (2)	0.0144 (19)	0.0015 (17)	0.0008 (17)
C27A	0.063 (3)	0.059 (3)	0.030 (3)	0.038 (3)	0.017 (2)	0.009 (2)
C28A	0.098 (5)	0.073 (4)	0.045 (3)	0.067 (4)	0.024 (3)	0.018 (3)
C29A	0.084 (4)	0.052 (3)	0.039 (3)	0.047 (3)	0.008 (3)	0.010 (2)
C30A	0.063 (3)	0.049 (3)	0.032 (3)	0.030 (3)	0.012 (2)	0.010 (2)
C31A	0.057 (3)	0.046 (3)	0.025 (2)	0.031 (2)	0.009 (2)	0.005 (2)
Au1B	0.03466 (10)	0.03069 (9)	0.02350 (9)	0.01670 (8)	0.00744 (7)	0.00507 (7)
Cl1B	0.0784 (9)	0.0528 (7)	0.0262 (6)	0.0337 (7)	0.0169 (6)	0.0079 (5)
P1B	0.0259 (5)	0.0279 (5)	0.0226 (5)	0.0135 (4)	0.0054 (4)	0.0060 (4)
N1B	0.026 (2)	0.055 (3)	0.038 (2)	0.0140 (19)	0.0013 (17)	−0.0079 (19)
C1B	0.021 (2)	0.039 (3)	0.038 (3)	0.0006 (19)	0.0084 (19)	−0.003 (2)
C2B	0.024 (2)	0.043 (3)	0.034 (3)	0.006 (2)	0.0034 (19)	−0.001 (2)
C3B	0.034 (3)	0.041 (3)	0.033 (3)	0.004 (2)	0.008 (2)	−0.001 (2)
C4B	0.056 (3)	0.037 (3)	0.043 (3)	0.002 (2)	0.013 (3)	−0.003 (2)
C5B	0.060 (3)	0.040 (3)	0.043 (3)	0.005 (3)	0.014 (3)	0.010 (2)
C6B	0.039 (3)	0.040 (3)	0.031 (3)	−0.002 (2)	0.009 (2)	−0.001 (2)
C7B	0.037 (3)	0.051 (3)	0.039 (3)	0.017 (2)	−0.003 (2)	−0.008 (2)
C8B	0.055 (4)	0.072 (4)	0.083 (5)	0.028 (3)	−0.029 (3)	−0.009 (4)
C9B	0.069 (4)	0.068 (4)	0.065 (4)	0.036 (3)	0.023 (3)	0.023 (3)
C10B	0.064 (4)	0.037 (3)	0.034 (3)	0.003 (3)	0.009 (2)	0.006 (2)
C11B	0.068 (5)	0.260 (12)	0.049 (4)	0.099 (6)	−0.007 (3)	−0.011 (5)
C12B	0.077 (5)	0.190 (8)	0.050 (4)	0.086 (5)	0.031 (3)	0.040 (5)
C13B	0.023 (2)	0.036 (2)	0.027 (2)	0.0100 (19)	0.0042 (17)	0.0094 (18)
C14B	0.028 (2)	0.036 (2)	0.024 (2)	0.0171 (19)	0.0051 (17)	0.0071 (18)
C15B	0.025 (2)	0.031 (2)	0.022 (2)	0.0135 (18)	0.0048 (16)	0.0084 (17)
C16B	0.032 (2)	0.035 (2)	0.036 (3)	0.014 (2)	0.0080 (19)	0.001 (2)
C17B	0.046 (3)	0.054 (3)	0.050 (3)	0.027 (3)	0.013 (2)	−0.006 (3)
C18B	0.035 (3)	0.067 (4)	0.057 (3)	0.028 (3)	0.015 (2)	−0.004 (3)
C19B	0.026 (2)	0.052 (3)	0.040 (3)	0.017 (2)	0.005 (2)	0.006 (2)
C20B	0.029 (2)	0.028 (2)	0.032 (2)	0.0123 (18)	0.0034 (18)	0.0096 (18)
C21B	0.051 (3)	0.057 (3)	0.053 (3)	0.037 (3)	0.019 (2)	0.023 (3)
C22B	0.065 (4)	0.058 (3)	0.079 (4)	0.046 (3)	0.022 (3)	0.031 (3)
C23B	0.053 (3)	0.046 (3)	0.065 (4)	0.023 (3)	0.006 (3)	0.030 (3)
C24B	0.046 (3)	0.038 (3)	0.037 (3)	0.009 (2)	0.004 (2)	0.015 (2)
C25B	0.033 (2)	0.030 (2)	0.034 (2)	0.0118 (19)	0.0065 (19)	0.0083 (19)
C26B	0.022 (2)	0.030 (2)	0.025 (2)	0.0108 (17)	0.0065 (16)	0.0069 (17)
C27B	0.030 (2)	0.038 (3)	0.029 (2)	0.010 (2)	−0.0046 (18)	0.008 (2)
C28B	0.037 (3)	0.035 (3)	0.038 (3)	0.010 (2)	0.003 (2)	0.013 (2)
C29B	0.027 (2)	0.036 (3)	0.037 (3)	0.004 (2)	0.0041 (19)	0.006 (2)
C30B	0.033 (2)	0.043 (3)	0.028 (2)	0.011 (2)	−0.0031 (19)	0.008 (2)
C31B	0.031 (2)	0.036 (2)	0.029 (2)	0.013 (2)	0.0077 (18)	0.0131 (19)
Cl4	0.197 (7)	0.065 (3)	0.110 (4)	0.052 (4)	0.059 (4)	0.022 (3)
Cl3	0.184 (8)	0.077 (4)	0.261 (9)	0.082 (5)	0.144 (7)	0.060 (6)
Cl2	0.116 (4)	0.081 (3)	0.322 (10)	0.014 (3)	−0.059 (5)	0.076 (5)
C32	0.079 (9)	0.068 (9)	0.101 (11)	0.045 (7)	0.049 (9)	0.044 (8)

### Geometric parameters (Å, º)

**Table:**

Au1A—P1A	2.2354 (10)	P1B—C26B	1.811 (4)
Au1A—Cl1A	2.2783 (11)	P1B—C20B	1.814 (4)
P1A—C26A	1.807 (4)	P1B—C15B	1.828 (4)
P1A—C20A	1.824 (4)	N1B—C13B	1.258 (5)
P1A—C15A	1.835 (4)	N1B—C1B	1.435 (6)
N1A—C13A	1.236 (6)	C1B—C2B	1.404 (6)
N1A—C1A	1.442 (6)	C1B—C6B	1.411 (7)
C1A—C2A	1.399 (6)	C2B—C3B	1.389 (7)
C1A—C6A	1.400 (7)	C2B—C7B	1.514 (7)
C2A—C3A	1.383 (6)	C3B—C4B	1.369 (7)
C2A—C7A	1.534 (7)	C3B—H3B	0.9500
C3A—C4A	1.377 (7)	C4B—C5B	1.388 (7)
C3A—H3A	0.9500	C4B—H4B	0.9500
C4A—C5A	1.373 (7)	C5B—C6B	1.395 (7)
C4A—H4A	0.9500	C5B—H5B	0.9500
C5A—C6A	1.387 (7)	C6B—C10B	1.520 (7)
C5A—H5A	0.9500	C7B—C9B	1.520 (7)
C6A—C10A	1.558 (8)	C7B—C8B	1.538 (7)
C7A—C8A	1.476 (8)	C7B—H7B	1.0000
C7A—C9A	1.518 (9)	C8B—H8BA	0.9800
C7A—H7A	1.0000	C8B—H8BB	0.9800
C8A—H8AA	0.9800	C8B—H8BC	0.9800
C8A—H8AB	0.9800	C9B—H9BA	0.9800
C8A—H8AC	0.9800	C9B—H9BB	0.9800
C9A—H9AA	0.9800	C9B—H9BC	0.9800
C9A—H9AB	0.9800	C10B—C12B	1.490 (8)
C9A—H9AC	0.9800	C10B—C11B	1.504 (9)
C10A—C11A	1.460 (11)	C10B—H10B	1.0000
C10A—C12A	1.499 (10)	C11B—H11A	0.9800
C10A—H10A	1.0000	C11B—H11B	0.9800
C11A—H11D	0.9800	C11B—H11C	0.9800
C11A—H11E	0.9800	C12B—H12A	0.9800
C11A—H11F	0.9800	C12B—H12B	0.9800
C12A—H12D	0.9800	C12B—H12C	0.9800
C12A—H12E	0.9800	C13B—C14B	1.487 (6)
C12A—H12F	0.9800	C13B—H13B	0.9500
C13A—C14A	1.497 (6)	C14B—C19B	1.396 (6)
C13A—H13A	0.9500	C14B—C15B	1.404 (6)
C14A—C19A	1.385 (6)	C15B—C16B	1.392 (6)
C14A—C15A	1.409 (6)	C16B—C17B	1.388 (6)
C15A—C16A	1.393 (5)	C16B—H16B	0.9500
C16A—C17A	1.390 (6)	C17B—C18B	1.376 (7)
C16A—H16A	0.9500	C17B—H17B	0.9500
C17A—C18A	1.375 (6)	C18B—C19B	1.382 (7)
C17A—H17A	0.9500	C18B—H18B	0.9500
C18A—C19A	1.387 (6)	C19B—H19B	0.9500
C18A—H18A	0.9500	C20B—C21B	1.385 (6)
C19A—H19A	0.9500	C20B—C25B	1.401 (6)
C20A—C21A	1.378 (6)	C21B—C22B	1.386 (7)
C20A—C25A	1.383 (6)	C21B—H21B	0.9500
C21A—C22A	1.385 (6)	C22B—C23B	1.369 (8)
C21A—H21A	0.9500	C22B—H22B	0.9500
C22A—C23A	1.375 (7)	C23B—C24B	1.376 (7)
C22A—H22A	0.9500	C23B—H23B	0.9500
C23A—C24A	1.385 (6)	C24B—C25B	1.382 (6)
C23A—H23A	0.9500	C24B—H24B	0.9500
C24A—C25A	1.386 (6)	C25B—H25B	0.9500
C24A—H24A	0.9500	C26B—C31B	1.391 (6)
C25A—H25A	0.9500	C26B—C27B	1.394 (6)
C26A—C27A	1.384 (6)	C27B—C28B	1.385 (6)
C26A—C31A	1.393 (6)	C27B—H27B	0.9500
C27A—C28A	1.377 (7)	C28B—C29B	1.380 (6)
C27A—H27A	0.9500	C28B—H28B	0.9500
C28A—C29A	1.375 (7)	C29B—C30B	1.378 (6)
C28A—H28A	0.9500	C29B—H29B	0.9500
C29A—C30A	1.373 (7)	C30B—C31B	1.390 (6)
C29A—H29A	0.9500	C30B—H30B	0.9500
C30A—C31A	1.383 (6)	C31B—H31B	0.9500
C30A—H30A	0.9500	Cl4—C32	1.903 (16)
C31A—H31A	0.9500	Cl3—C32	1.739 (14)
Au1B—P1B	2.2267 (10)	Cl2—C32	1.621 (15)
Au1B—Cl1B	2.2707 (11)	C32—H32	1.0000
			
P1A—Au1A—Cl1A	177.20 (4)	C20B—P1B—C15B	104.38 (19)
C26A—P1A—C20A	105.25 (19)	C26B—P1B—Au1B	110.11 (13)
C26A—P1A—C15A	105.82 (19)	C20B—P1B—Au1B	115.53 (15)
C20A—P1A—C15A	105.36 (18)	C15B—P1B—Au1B	113.46 (13)
C26A—P1A—Au1A	110.70 (13)	C13B—N1B—C1B	118.3 (4)
C20A—P1A—Au1A	116.83 (13)	C2B—C1B—C6B	121.7 (4)
C15A—P1A—Au1A	112.07 (13)	C2B—C1B—N1B	118.4 (5)
C13A—N1A—C1A	117.9 (4)	C6B—C1B—N1B	119.8 (4)
C2A—C1A—C6A	122.2 (4)	C3B—C2B—C1B	118.3 (5)
C2A—C1A—N1A	118.8 (4)	C3B—C2B—C7B	120.6 (4)
C6A—C1A—N1A	118.9 (4)	C1B—C2B—C7B	121.1 (4)
C3A—C2A—C1A	117.3 (4)	C4B—C3B—C2B	121.5 (5)
C3A—C2A—C7A	120.9 (4)	C4B—C3B—H3B	119.3
C1A—C2A—C7A	121.9 (4)	C2B—C3B—H3B	119.3
C4A—C3A—C2A	121.4 (5)	C3B—C4B—C5B	119.7 (5)
C4A—C3A—H3A	119.3	C3B—C4B—H4B	120.2
C2A—C3A—H3A	119.3	C5B—C4B—H4B	120.2
C5A—C4A—C3A	120.4 (5)	C4B—C5B—C6B	121.9 (5)
C5A—C4A—H4A	119.8	C4B—C5B—H5B	119.0
C3A—C4A—H4A	119.8	C6B—C5B—H5B	119.0
C4A—C5A—C6A	120.9 (5)	C5B—C6B—C1B	117.0 (4)
C4A—C5A—H5A	119.6	C5B—C6B—C10B	119.8 (5)
C6A—C5A—H5A	119.6	C1B—C6B—C10B	123.1 (5)
C5A—C6A—C1A	117.7 (5)	C2B—C7B—C9B	109.8 (4)
C5A—C6A—C10A	121.4 (5)	C2B—C7B—C8B	112.1 (4)
C1A—C6A—C10A	120.9 (4)	C9B—C7B—C8B	111.4 (5)
C8A—C7A—C9A	111.3 (5)	C2B—C7B—H7B	107.8
C8A—C7A—C2A	111.7 (5)	C9B—C7B—H7B	107.8
C9A—C7A—C2A	114.4 (5)	C8B—C7B—H7B	107.8
C8A—C7A—H7A	106.3	C7B—C8B—H8BA	109.5
C9A—C7A—H7A	106.3	C7B—C8B—H8BB	109.5
C2A—C7A—H7A	106.3	H8BA—C8B—H8BB	109.5
C7A—C8A—H8AA	109.5	C7B—C8B—H8BC	109.5
C7A—C8A—H8AB	109.5	H8BA—C8B—H8BC	109.5
H8AA—C8A—H8AB	109.5	H8BB—C8B—H8BC	109.5
C7A—C8A—H8AC	109.5	C7B—C9B—H9BA	109.5
H8AA—C8A—H8AC	109.5	C7B—C9B—H9BB	109.5
H8AB—C8A—H8AC	109.5	H9BA—C9B—H9BB	109.5
C7A—C9A—H9AA	109.5	C7B—C9B—H9BC	109.5
C7A—C9A—H9AB	109.5	H9BA—C9B—H9BC	109.5
H9AA—C9A—H9AB	109.5	H9BB—C9B—H9BC	109.5
C7A—C9A—H9AC	109.5	C12B—C10B—C11B	107.9 (5)
H9AA—C9A—H9AC	109.5	C12B—C10B—C6B	110.2 (5)
H9AB—C9A—H9AC	109.5	C11B—C10B—C6B	114.0 (5)
C11A—C10A—C12A	111.7 (7)	C12B—C10B—H10B	108.2
C11A—C10A—C6A	108.8 (7)	C11B—C10B—H10B	108.2
C12A—C10A—C6A	114.0 (5)	C6B—C10B—H10B	108.2
C11A—C10A—H10A	107.4	C10B—C11B—H11A	109.5
C12A—C10A—H10A	107.4	C10B—C11B—H11B	109.5
C6A—C10A—H10A	107.4	H11A—C11B—H11B	109.5
C10A—C11A—H11D	109.5	C10B—C11B—H11C	109.5
C10A—C11A—H11E	109.5	H11A—C11B—H11C	109.5
H11D—C11A—H11E	109.5	H11B—C11B—H11C	109.5
C10A—C11A—H11F	109.5	C10B—C12B—H12A	109.5
H11D—C11A—H11F	109.5	C10B—C12B—H12B	109.5
H11E—C11A—H11F	109.5	H12A—C12B—H12B	109.5
C10A—C12A—H12D	109.5	C10B—C12B—H12C	109.5
C10A—C12A—H12E	109.5	H12A—C12B—H12C	109.5
H12D—C12A—H12E	109.5	H12B—C12B—H12C	109.5
C10A—C12A—H12F	109.5	N1B—C13B—C14B	121.9 (4)
H12D—C12A—H12F	109.5	N1B—C13B—H13B	119.1
H12E—C12A—H12F	109.5	C14B—C13B—H13B	119.1
N1A—C13A—C14A	122.4 (4)	C19B—C14B—C15B	118.7 (4)
N1A—C13A—H13A	118.8	C19B—C14B—C13B	119.6 (4)
C14A—C13A—H13A	118.8	C15B—C14B—C13B	121.7 (4)
C19A—C14A—C15A	119.1 (4)	C16B—C15B—C14B	119.7 (4)
C19A—C14A—C13A	120.5 (4)	C16B—C15B—P1B	119.6 (3)
C15A—C14A—C13A	120.4 (4)	C14B—C15B—P1B	120.6 (3)
C16A—C15A—C14A	119.5 (4)	C17B—C16B—C15B	120.9 (4)
C16A—C15A—P1A	119.3 (3)	C17B—C16B—H16B	119.6
C14A—C15A—P1A	121.1 (3)	C15B—C16B—H16B	119.6
C17A—C16A—C15A	120.0 (4)	C18B—C17B—C16B	119.2 (5)
C17A—C16A—H16A	120.0	C18B—C17B—H17B	120.4
C15A—C16A—H16A	120.0	C16B—C17B—H17B	120.4
C18A—C17A—C16A	120.6 (4)	C17B—C18B—C19B	120.9 (4)
C18A—C17A—H17A	119.7	C17B—C18B—H18B	119.5
C16A—C17A—H17A	119.7	C19B—C18B—H18B	119.5
C17A—C18A—C19A	119.7 (4)	C18B—C19B—C14B	120.6 (4)
C17A—C18A—H18A	120.1	C18B—C19B—H19B	119.7
C19A—C18A—H18A	120.1	C14B—C19B—H19B	119.7
C14A—C19A—C18A	121.1 (4)	C21B—C20B—C25B	119.0 (4)
C14A—C19A—H19A	119.5	C21B—C20B—P1B	119.6 (3)
C18A—C19A—H19A	119.5	C25B—C20B—P1B	121.4 (3)
C21A—C20A—C25A	120.0 (4)	C20B—C21B—C22B	120.1 (5)
C21A—C20A—P1A	119.4 (3)	C20B—C21B—H21B	119.9
C25A—C20A—P1A	120.4 (3)	C22B—C21B—H21B	119.9
C20A—C21A—C22A	119.7 (4)	C23B—C22B—C21B	120.6 (5)
C20A—C21A—H21A	120.1	C23B—C22B—H22B	119.7
C22A—C21A—H21A	120.1	C21B—C22B—H22B	119.7
C23A—C22A—C21A	120.4 (4)	C22B—C23B—C24B	120.0 (5)
C23A—C22A—H22A	119.8	C22B—C23B—H23B	120.0
C21A—C22A—H22A	119.8	C24B—C23B—H23B	120.0
C22A—C23A—C24A	120.1 (4)	C23B—C24B—C25B	120.4 (5)
C22A—C23A—H23A	120.0	C23B—C24B—H24B	119.8
C24A—C23A—H23A	120.0	C25B—C24B—H24B	119.8
C23A—C24A—C25A	119.5 (4)	C24B—C25B—C20B	120.0 (4)
C23A—C24A—H24A	120.3	C24B—C25B—H25B	120.0
C25A—C24A—H24A	120.3	C20B—C25B—H25B	120.0
C20A—C25A—C24A	120.2 (4)	C31B—C26B—C27B	119.0 (4)
C20A—C25A—H25A	119.9	C31B—C26B—P1B	122.2 (3)
C24A—C25A—H25A	119.9	C27B—C26B—P1B	118.8 (3)
C27A—C26A—C31A	118.5 (4)	C28B—C27B—C26B	120.6 (4)
C27A—C26A—P1A	119.4 (3)	C28B—C27B—H27B	119.7
C31A—C26A—P1A	122.1 (3)	C26B—C27B—H27B	119.7
C28A—C27A—C26A	121.1 (4)	C29B—C28B—C27B	119.7 (4)
C28A—C27A—H27A	119.5	C29B—C28B—H28B	120.2
C26A—C27A—H27A	119.5	C27B—C28B—H28B	120.2
C29A—C28A—C27A	119.6 (5)	C30B—C29B—C28B	120.5 (4)
C29A—C28A—H28A	120.2	C30B—C29B—H29B	119.7
C27A—C28A—H28A	120.2	C28B—C29B—H29B	119.7
C30A—C29A—C28A	120.6 (5)	C29B—C30B—C31B	120.0 (4)
C30A—C29A—H29A	119.7	C29B—C30B—H30B	120.0
C28A—C29A—H29A	119.7	C31B—C30B—H30B	120.0
C29A—C30A—C31A	119.8 (5)	C30B—C31B—C26B	120.2 (4)
C29A—C30A—H30A	120.1	C30B—C31B—H31B	119.9
C31A—C30A—H30A	120.1	C26B—C31B—H31B	119.9
C30A—C31A—C26A	120.4 (4)	Cl2—C32—Cl3	125.5 (10)
C30A—C31A—H31A	119.8	Cl2—C32—Cl4	107.2 (9)
C26A—C31A—H31A	119.8	Cl3—C32—Cl4	95.9 (7)
P1B—Au1B—Cl1B	178.54 (4)	Cl2—C32—H32	108.9
C26B—P1B—C20B	106.29 (19)	Cl3—C32—H32	108.9
C26B—P1B—C15B	106.40 (18)	Cl4—C32—H32	108.9
			
C13A—N1A—C1A—C2A	−90.8 (5)	C13B—N1B—C1B—C2B	−119.9 (5)
C13A—N1A—C1A—C6A	93.0 (6)	C13B—N1B—C1B—C6B	65.0 (6)
C6A—C1A—C2A—C3A	3.6 (7)	C6B—C1B—C2B—C3B	−2.1 (6)
N1A—C1A—C2A—C3A	−172.4 (4)	N1B—C1B—C2B—C3B	−177.1 (4)
C6A—C1A—C2A—C7A	−176.1 (5)	C6B—C1B—C2B—C7B	−180.0 (4)
N1A—C1A—C2A—C7A	7.9 (7)	N1B—C1B—C2B—C7B	5.0 (6)
C1A—C2A—C3A—C4A	−1.8 (7)	C1B—C2B—C3B—C4B	0.7 (7)
C7A—C2A—C3A—C4A	177.8 (5)	C7B—C2B—C3B—C4B	178.6 (4)
C2A—C3A—C4A—C5A	−0.5 (8)	C2B—C3B—C4B—C5B	0.3 (7)
C3A—C4A—C5A—C6A	1.3 (9)	C3B—C4B—C5B—C6B	−0.1 (8)
C4A—C5A—C6A—C1A	0.4 (8)	C4B—C5B—C6B—C1B	−1.2 (7)
C4A—C5A—C6A—C10A	−178.5 (6)	C4B—C5B—C6B—C10B	175.4 (5)
C2A—C1A—C6A—C5A	−2.9 (8)	C2B—C1B—C6B—C5B	2.3 (7)
N1A—C1A—C6A—C5A	173.1 (5)	N1B—C1B—C6B—C5B	177.2 (4)
C2A—C1A—C6A—C10A	176.0 (5)	C2B—C1B—C6B—C10B	−174.1 (4)
N1A—C1A—C6A—C10A	−8.0 (8)	N1B—C1B—C6B—C10B	0.8 (7)
C3A—C2A—C7A—C8A	93.8 (7)	C3B—C2B—C7B—C9B	−75.9 (5)
C1A—C2A—C7A—C8A	−86.6 (7)	C1B—C2B—C7B—C9B	101.9 (5)
C3A—C2A—C7A—C9A	−33.8 (8)	C3B—C2B—C7B—C8B	48.4 (6)
C1A—C2A—C7A—C9A	145.9 (6)	C1B—C2B—C7B—C8B	−133.7 (5)
C5A—C6A—C10A—C11A	−95.5 (9)	C5B—C6B—C10B—C12B	−85.3 (7)
C1A—C6A—C10A—C11A	85.6 (9)	C1B—C6B—C10B—C12B	91.0 (6)
C5A—C6A—C10A—C12A	29.8 (10)	C5B—C6B—C10B—C11B	36.2 (7)
C1A—C6A—C10A—C12A	−149.1 (6)	C1B—C6B—C10B—C11B	−147.5 (6)
C1A—N1A—C13A—C14A	−179.9 (4)	C1B—N1B—C13B—C14B	−174.4 (4)
N1A—C13A—C14A—C19A	−3.6 (6)	N1B—C13B—C14B—C19B	13.4 (6)
N1A—C13A—C14A—C15A	176.0 (4)	N1B—C13B—C14B—C15B	−168.9 (4)
C19A—C14A—C15A—C16A	1.2 (6)	C19B—C14B—C15B—C16B	−0.9 (6)
C13A—C14A—C15A—C16A	−178.5 (4)	C13B—C14B—C15B—C16B	−178.6 (4)
C19A—C14A—C15A—P1A	−175.3 (3)	C19B—C14B—C15B—P1B	177.5 (3)
C13A—C14A—C15A—P1A	5.0 (5)	C13B—C14B—C15B—P1B	−0.2 (5)
C26A—P1A—C15A—C16A	4.0 (4)	C26B—P1B—C15B—C16B	−4.7 (4)
C20A—P1A—C15A—C16A	115.2 (3)	C20B—P1B—C15B—C16B	107.5 (4)
Au1A—P1A—C15A—C16A	−116.7 (3)	Au1B—P1B—C15B—C16B	−125.9 (3)
C26A—P1A—C15A—C14A	−179.5 (3)	C26B—P1B—C15B—C14B	176.9 (3)
C20A—P1A—C15A—C14A	−68.3 (4)	C20B—P1B—C15B—C14B	−70.9 (4)
Au1A—P1A—C15A—C14A	59.8 (3)	Au1B—P1B—C15B—C14B	55.7 (3)
C14A—C15A—C16A—C17A	−0.9 (6)	C14B—C15B—C16B—C17B	0.9 (7)
P1A—C15A—C16A—C17A	175.7 (3)	P1B—C15B—C16B—C17B	−177.5 (4)
C15A—C16A—C17A—C18A	0.0 (7)	C15B—C16B—C17B—C18B	−0.1 (8)
C16A—C17A—C18A—C19A	0.6 (7)	C16B—C17B—C18B—C19B	−0.7 (8)
C15A—C14A—C19A—C18A	−0.6 (6)	C17B—C18B—C19B—C14B	0.6 (8)
C13A—C14A—C19A—C18A	179.1 (4)	C15B—C14B—C19B—C18B	0.2 (7)
C17A—C18A—C19A—C14A	−0.3 (7)	C13B—C14B—C19B—C18B	177.9 (4)
C26A—P1A—C20A—C21A	−86.0 (4)	C26B—P1B—C20B—C21B	−92.6 (4)
C15A—P1A—C20A—C21A	162.4 (3)	C15B—P1B—C20B—C21B	155.1 (4)
Au1A—P1A—C20A—C21A	37.2 (4)	Au1B—P1B—C20B—C21B	29.8 (4)
C26A—P1A—C20A—C25A	90.8 (4)	C26B—P1B—C20B—C25B	84.7 (4)
C15A—P1A—C20A—C25A	−20.8 (4)	C15B—P1B—C20B—C25B	−27.6 (4)
Au1A—P1A—C20A—C25A	−145.9 (3)	Au1B—P1B—C20B—C25B	−152.9 (3)
C25A—C20A—C21A—C22A	−0.2 (7)	C25B—C20B—C21B—C22B	−0.2 (7)
P1A—C20A—C21A—C22A	176.6 (4)	P1B—C20B—C21B—C22B	177.1 (4)
C20A—C21A—C22A—C23A	0.2 (7)	C20B—C21B—C22B—C23B	0.4 (9)
C21A—C22A—C23A—C24A	−0.3 (7)	C21B—C22B—C23B—C24B	−0.3 (9)
C22A—C23A—C24A—C25A	0.4 (7)	C22B—C23B—C24B—C25B	0.1 (8)
C21A—C20A—C25A—C24A	0.3 (6)	C23B—C24B—C25B—C20B	0.1 (7)
P1A—C20A—C25A—C24A	−176.5 (3)	C21B—C20B—C25B—C24B	0.0 (7)
C23A—C24A—C25A—C20A	−0.4 (7)	P1B—C20B—C25B—C24B	−177.3 (3)
C20A—P1A—C26A—C27A	159.0 (4)	C20B—P1B—C26B—C31B	−5.3 (4)
C15A—P1A—C26A—C27A	−89.7 (4)	C15B—P1B—C26B—C31B	105.5 (4)
Au1A—P1A—C26A—C27A	31.9 (4)	Au1B—P1B—C26B—C31B	−131.2 (3)
C20A—P1A—C26A—C31A	−20.9 (4)	C20B—P1B—C26B—C27B	173.3 (3)
C15A—P1A—C26A—C31A	90.4 (4)	C15B—P1B—C26B—C27B	−75.9 (4)
Au1A—P1A—C26A—C31A	−148.0 (3)	Au1B—P1B—C26B—C27B	47.5 (4)
C31A—C26A—C27A—C28A	−1.1 (7)	C31B—C26B—C27B—C28B	−1.6 (7)
P1A—C26A—C27A—C28A	178.9 (4)	P1B—C26B—C27B—C28B	179.7 (4)
C26A—C27A—C28A—C29A	2.0 (9)	C26B—C27B—C28B—C29B	1.5 (7)
C27A—C28A—C29A—C30A	−1.3 (9)	C27B—C28B—C29B—C30B	−0.2 (7)
C28A—C29A—C30A—C31A	−0.2 (8)	C28B—C29B—C30B—C31B	−0.9 (7)
C29A—C30A—C31A—C26A	1.1 (8)	C29B—C30B—C31B—C26B	0.7 (7)
C27A—C26A—C31A—C30A	−0.4 (7)	C27B—C26B—C31B—C30B	0.6 (6)
P1A—C26A—C31A—C30A	179.5 (4)	P1B—C26B—C31B—C30B	179.2 (3)
